# Arterial aneurysmal sac-ureteral fistula after aortic and common iliac aneurysmal repair: A case report

**DOI:** 10.1016/j.eucr.2023.102560

**Published:** 2023-09-11

**Authors:** Noriyuki Makita, Masashi Kubota, Koji Inoue, Mutsushi Kawakita

**Affiliations:** Department of Urology, Kobe City Medical Centre General Hospital, 2-1-1 Minatojima-Minamimachi, Chuo-ku, Kobe, 650-0047, Japan

**Keywords:** Fistula, Ureter, Aortic aneurysm, Ureterocutaneostomy

## Abstract

An arterioureteral fistula is the communication between the ureter and a major artery, such as the common iliac artery or aorta. Here, we report a case of a fistula between the ureter and the common iliac arterial aneurysmal sac following abdominal aortic aneurysmal repair in a 72-year-old man. He reported acute-onset abdominal pain on postoperative day 8, and computed tomography revealed a fistula. Ureterocutaneostomy was performed to prevent urine inflow into the aneurysm and to preserve kidney function.

## 1 Introduction

An arterioureteral fistula refers to the communication between the ureter and a major artery, such as the common iliac artery or aorta. Although rare, this condition can be life-threatening as a result of massive bleeding. We describe a case of a fistula between the right common iliac aneurysmal sac and right ureter after aortic and common iliac arterial aneurysmal repair.

## 2 Case presentation

**Fig. 1:**
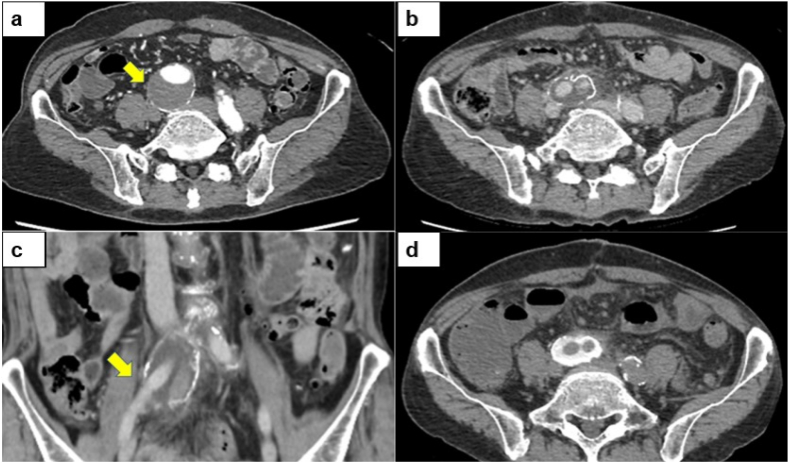
(a) Preoperative contrast-enhanced CT image showing the right common iliac aneurysmal sac adjacent to the right ureter (arrow). (b), (c) Postoperative contrast CT image showing fluid-filled right common iliac aneurysmal sac. The arrow indicates the right ureter. (d) Plain CT revealed accumulation of contrast medium within the right common iliac aneurysmal sac.

A 72-year-old man was referred to the cardiovascular department of our hospital for abdominal aortic aneurysm repair (Fig. 1-a). The patient had a history of rheumatoid arthritis which was being treated by prednisolone (5mg).

The patient underwent repair of an abdominal aortic aneurysm and bilateral common iliac artery aneurysms. Intraoperatively, the distal side of the Y-graft was anastomosed with the right external iliac artery, left common iliac artery, and proximal side with the nonaneurysmal infrarenal aorta. The wall of the aneurysm was sutured and the graft was wrapped.

On postoperative day 8, the patient complained of acute right lower abdominal pain. Contrast-enhanced computed tomography (CT) revealed mild right hydronephrosis with ureteral dilation to the level crossing the right common iliac aneurysmal sac (Fig. 1-b, c). The following day, the patient was referred to the urology department.

The patient's creatinine was 1.28mg/dL, which was elevated from his baseline of 0.7 mg/dL. Plain CT demonstrated the contrast medium administered the day before in the aneurysmal sac (Fig. 1-d). Furthermore, some of the contrast medium leaked into the abdominal cavity. We diagnosed a fistula between the right ureter and the right common iliac arterial aneurysmal sac.

We attempted to place a ureteral stent; however, the guide wire did not cross the aneurysmal sac. Moreover, we could not place a nephrostomy tube because the hydronephrosis was mild. Finally, a ureterocutaneostomy was performed via a lumbar oblique incision on the same day.

The next day, the abdominal pain disappeared, and the creatinine level returned to a baseline of 0.7 mg/dL. We removed the ureteral stent from the cutaneous urostomy on postoperative day 14, and the patient was discharged on postoperative day 21. The patient has remained asymptomatic for 2 years without graft infection or aneurysmal enlargement, and the cutaneous ureterostomy has not needed stenting after discharge.

## 3 Discussion

Arterioureteral fistulas are rare, but often causes fatal conditions due to massive bleeding. The mortality rate is up to 13%, and treatment is often delayed due to difficulties in diagnosis.^1^

Approximately 85% of cases are strongly associated with treatment procedures, such as ureteral stenting, urinary deviation surgery, vascular abdominal surgery, and radiotherapy, among others.^2^

In the pathophysiology, the ureter and aneurysm come into contact with each other due to radiation or postoperative adhesion, and the pressure and inflammation with chronic pulsation of blood vessels cause necrosis. This risk may increase with chronic ureteral stenting or vascular graft replacement due to increased pressure and inflammatory changes.^3^

An arterial aneurysmal sac-ureteral fistula has no direct communication between the ureter and the arterial blood flow; therefore, there is little concern about massive bleeding. However, due to concerns about complications such as graft infection, erosion, and bleeding due to continued exposure of the graft to urine, some treatment is needed. To our knowledge, fistula formation between the ureter and the wall of the aneurysm after aneurysmal repair has been described in only one case report, and it was treated by performing a nephrectomy.^4^

In this case, the CT image showed that the ureter and aneurysmal wall were close to each other before the operation, and it was considered that pulsatile trauma caused chronic inflammation. In addition, blood flow to the aneurysmal wall might be reduced by aneurysm repair surgery, leading to necrosis, fistula formation.^5^ The delayed wound healing may also be associated with long-term oral steroid use.

The acute abdominal pain was attributed to urinary peritonitis, with a small amount of urine leaking into the abdominal cavity. We could not place a ureteral stent or perform a nephrostomy, and the patient did not desire a nephrectomy. The adhesion around the ureter and aneurysm was so severe that it was difficult to lyse. The ureter, which was not adherent, was far away from bladder, and we could not make ureteral reimplantation; therefore, ureterocutaneostomy was performed. As a result, we prevented urine leakage around the graft and preserved renal function.

## 4 Conclusion

We report a rare case of a fistula between the ureter and common iliac arterial aneurysmal sac after abdominal aortic aneurysmal repair. To prevent urine inflow into the aneurysm and preserve kidney function, ureterocutaneostomy was performed, and the procedure was uneventful.

## Financial support

This study did not receive any specific grant from funding agencies in the public, commercial, or not-for-profit sectors.

## Consent

Written informed consent was obtained from the patient.

## Declaration of conflicts of interest

None.
